# Effect of Passage Number of Conditioned Medium Collected from Equine Amniotic Fluid Mesenchymal Stem Cells: Porcine Oocyte Maturation and Embryo Development

**DOI:** 10.3390/ijms23126569

**Published:** 2022-06-12

**Authors:** Ahyoung Park, Hyun Ju Oh, Kukbin Ji, Eunha Miri Choi, Dongern Kim, Eunyoung Kim, Min Kyu Kim

**Affiliations:** 1 Division of Animal and Dairy Science, College of Agriculture and Life Science, Chungnam National University, Daejeon 34134, Korea; pay1030@naver.com (A.P.); newborn52020@gmail.com (H.J.O.); jkb0076@naver.com (K.J.); tarutaru4585@naver.com (E.M.C.); labmaster11@naver.com (D.K.); 2 MK Biotech Inc., 99 Daehak-ro, Yuseong-gu, Daejeon 34134, Korea; key@mkbiotech.co.kr

**Keywords:** porcine oocyte, amniotic fluid mesenchymal stem cell-conditioned medium, antioxidants, in vitro fertilization, passage

## Abstract

Oocyte in vitro maturation (IVM) is the most important first step in in vitro embryo production. One prerequisite for the success of IVM in oocytes is to provide a rich culture microenvironment that meets the nutritional needs of developing oocytes. We applied different equine amniotic fluid mesenchymal stem cell conditioned medium (eAFMSC-CM) from passages 7, 18, and 27 to porcine oocytes during IVM to determine its effects on oocyte development and subsequent embryo development, specifically. The eAFMSC-CM from passage 7 (eAFMSC-CMp7) has a considerable impact on 9 genes: BAX, BCL2, SOD2, NRF2, TNFAIP6, PTGS2, HAS2, Cx37, and Cx43, which are associated with cumulus cell mediated oocyte maturation. GSH levels and distribution of mitochondrial and cortical granules were significantly increased in oocytes incubated with eAFMSC-CMp7. In addition, catalase and superoxide dismutase activities were high after IVM 44 h with eAFMSC-CMp7. After in vitro fertilization, blastocyst quality was significantly increased in the eAFMSC-CMp7 group compared to control. Lastly, the antioxidant effect of eAFMSC-CMp7 substantially regulated the expression of apoptosis, pluripotency related genes and decreased autophagy activity in blastocysts. Taken together, this study demonstrated that the eAFMSC-CMp7 enhanced the cytoplasmic maturation of oocytes and subsequent embryonic development by generating high antioxidant activity.

## 1. Introduction

In vitro maturation (IVM) of oocytes is the most important first step in the production of embryos in vitro, and it is essential for biomedical studies and assisted reproduction techniques [1,2]. Despite the increasing number of studies on IVM application, only a few oocytes that mature in vitro develop into blastocysts, whereas the rate of embryonic development in several species such as pigs is much higher when oocytes are matured in vivo [3,4,5,6]. For the oocyte meiosis to be successful, chromosomal segregation during nuclear maturation and cytoplasmic maturation including organelle distribution are indispensable to enhance developmental competence [7,8]. Cytoplasmic oocyte maturation also affects the completion of oocyte nuclear maturation, fertilization, and early embryonic development [9,10]. However, the current porcine oocyte IVM system may be suboptimal for complete synchronization of nuclear and/or cytoplasmic maturation [11].

One prerequisite for the success of IVM in oocytes is the provision of a rich culture microenvironment in vitro that meets the nutritional needs of the developing oocyte. It has been reported that when conditioned medium (CM) is added to IVM, multiple growth factors/cytokines present in the CM positively regulate the mRNA/protein expression of COC compared to MSC co-culture [12]. CM can be stored frozen, which allows simple use by making aliquots of the medium [13]. In addition, IVM has been improved by adding CM into the culture medium to regulate the IVM microenvironment and support oocyte development [14,15,16]. The extracellular vesicles constituting the secretome, microvesicles, and exosome are secreted from MSCs into the medium, and the medium collected after culturing MSCs in serum-free culture conditions is called MSC-CM. Such media have anti-inflammatory, antioxidant, and anti-apoptotic properties [17]. Conditioned media also increase cell viability and antioxidant enzyme activity, while reducing reactive oxygen species (ROS) levels and apoptosis [18].

Amniotic fluid mesenchymal stem cells (AFMSCs) express both embryonic and adult stem cell markers and can be induced to differentiate into cell types derived from different germ layers, including cells of adipogenic, osteogenic, myogenic, endothelial, neuronal and hepatic lineages [19,20,21,22,23]. Extracellular vesicles from AFMSCs contain antioxidant enzymes such as superoxide dismutase (SOD) 1 [24] and can secrete multiple growth factors that might have antioxidant effects [25,26]. The advantage of equine amniotic fluid mesenchymal stem cells (eAFMSC) is that large numbers can be obtained from very small volumes of amniotic fluid [27]. They also contain various innate and adaptive immune cells derived from maternal, fetal, and placental tissues and are used for cell therapy [28]. However, the effects of eAFMSC on oocyte and embryo development that are important for protecting the fetus remain unknown.

Changes in MSCs that occur during long-term culture include increased numbers of morphologically evaluated senescent cells, decreased proliferation potential, and a loss of osteogenic differentiation potential [29,30]. Some stem cells undergo senescence, which is characterized by permanent growth arrest, resistance to apoptosis, macromolecular damage, and altered metabolism [31,32]. Recently, equine bone marrow-derived MSCs demonstrated that an increase in passage number (from P3 to P7) in the cell culture did not have any significant effect on the immunoprivilege of MSC. It was also reported that there was no difference in doubling time and levels of the pro and anti-apoptotic proteins between passages 3 and 7 of equine MSCs [33]. In our preliminary study, it was confirmed that the doubling time increased along with passage of equine MSCs and the doubling time gradually increased for each 0–10, 11–20, and 21–30 passages. Therefore, based on these previous studies, the present study was designed for equine MSCs of passage 18 and passage 27, which differed by 10 passages based on passage 7. Until now, the number of passages is known to affect the culture of MSCs, but the effects of the passage numbers on oocytes and cumulus cells (CCs) in IVM are uncertain.

Here, we hypothesized that supplementation with eAFMSC-CM during IVM would influence oocyte quality and subsequent developmental competence by improving the microenvironment, and that the number of passages would affect the results. We aimed to determine the degree of cumulus expansion and IVM of porcine oocytes cultured with eAFMSC-CM from different numbers of passages, antioxidant enzymes in eAFMSC-CM, and embryo development after IVF in eAFMSC-CM from the optimal number of passages.

## 2. Results

### 2.1. Effects of eAFMSC-CM Passage Numbers on Cumulus Expansion during IVM (Experiment 1)

**Figure 1:**
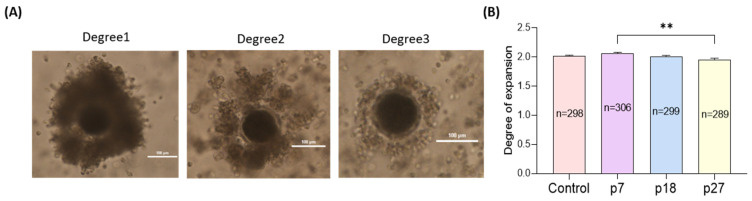
Effects of different passages of eAFMSC-CM on cumulus expansion. (**A**) The degree of cumulus cell expansion was classified into three grades: Degree 1 (no cumulus expansion), Degree 2 (moderate expansion), and Degree 3 (full expansion). (**B**) Average the degree of cumulus cell expansion. p7; eAFMSC-CMp7, p18; eAFMSC-CMp18, p27; eAFMSC-CMp27. The asterisks represent significantly different when compared to the control (**: *p* < 0.01). Scale bar = 100 μm.

We evaluated the effects of eAFMSC-CM from passages 7, 18, and 27 (p7, p18, and p27) on cumulus cell expansion after IVM for 44 h (Figure 1). Cumulus expansion was significantly greater in the eAFMSC-CMp7 supplemented medium, than in the control, p18, and p27 groups groups (2.06 ± 0.02 vs. 2.02 ± 0.01, 2.01 ± 0.01, and 1.96 ± 0.02, respectively, *p* < 0.05; Figure 1A,B).

**Figure 2:**
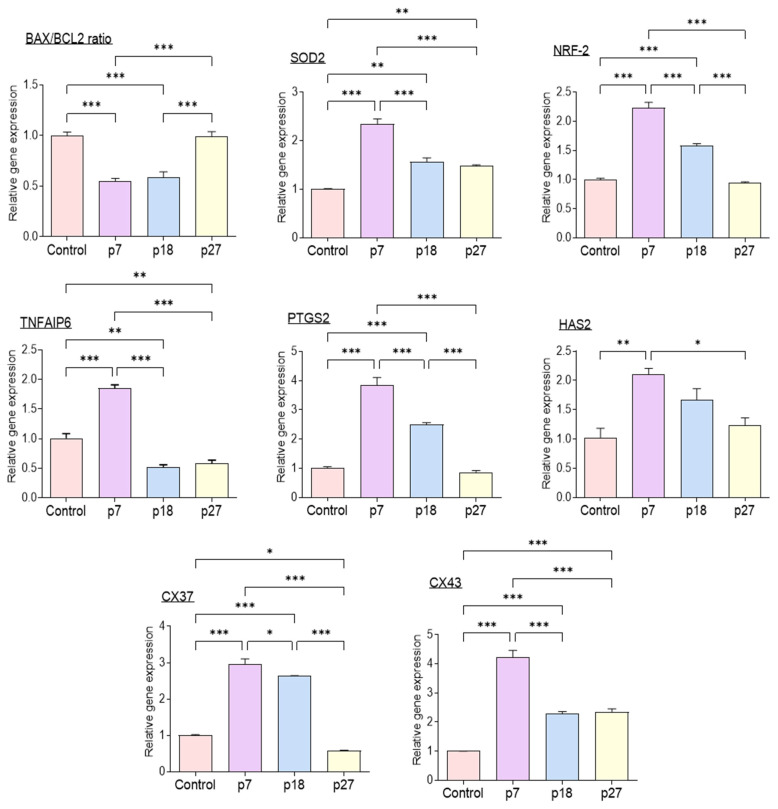
Relative expression of apoptosis-related genes (BAX, BCL2), antioxidant enzyme relative genes (SOD2 and Nrf-2), cumulus expansion related genes (HAS2, TNFAIP6 and PTGS2), and cumulus gap junction related genes (Cx37 and Cx43) in porcine cumulus cells. The asterisks represent significantly different when compared to the control (*: *p* < 0.05, **: *p* < 0.01, ***: *p* < 0.001,). Scale bar = 100 μm. Bax; BCL2 Associated X, Bcl2; B-cell lymphoma 2, HAS2; hyaluronan synthase 2, TNFAIP6; tumor necrosis factor α-induced protein 6, PTGS2; prostaglandin-endoperoxide synthase 2, Nrf-2; nuclear factor erythroid 2–related factor 2, SOD2; Superoxide dismutase 2, CX37; Connexin 37, CX43; Connexin 43.

Figure 2 shows the relative expression of genes associated with apoptosis, antioxidant enzymes, cumulus cell expansion, and gap junctions in porcine cumulus cells analyzed using qPCR. The BAX/BCL-2 ratio was significantly lower in the eAFMSC-CMp7 group than in the control (*p* < 0.05). The expression of antioxidant-related genes, SOD2 and NRF-2, was significantly increased in the eAFMSC-CMp7 group compared to the control and other eAFMSC-CM groups (*p* < 0.05). Furthermore, the expression of genes associated with cumulus expansion, namely tumor necrosis alpha induced protein 6 (TNFAIP6) and prostaglandin-endoperoxide synthase 2 (PTGS2), was significantly increased in the eAFMSC-CMp7, compared to all other groups (*p* < 0.05). Levels of Cx43 transcripts were also significantly increased in eAFMSC-CMp7 compared to the other groups (*p* < 0.05). 

### 2.2. Effects of eAFMSC-CM Passages during IVM on Oocyte Cytoplasmic Maturation (Experiment 2)

**Figure 3:**
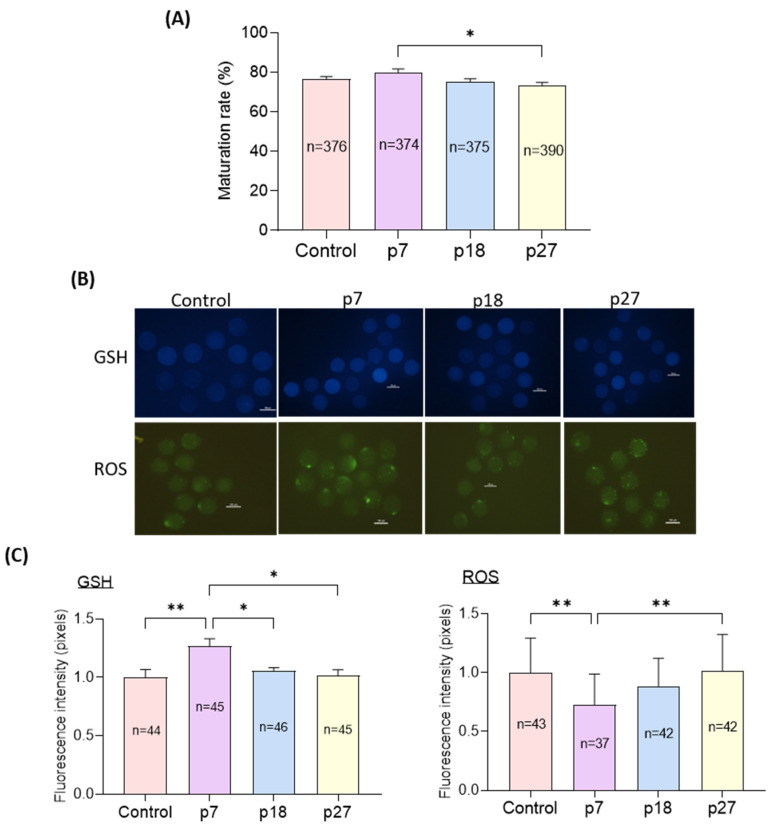
Effects of different passages of eAFMSC-CM on oocyte cytoplasmic maturation. (**A**) Rates of matured oocytes. (**B**) Images of oocytes stained with CMF2HC and DCFHDA. (**C**) Quantification of the intracellular GSH and ROS levels in MII oocytes. The asterisks represent significantly different when compared to the control (*: *p* < 0.05, **: *p* < 0.01). Scale bar = 100 μm.

We evaluated the effects of eAFMSC-CM from passages 7, 18, and 27 (p7, p18, and p27, respectively) on oocyte cytoplasmic maturation after IVM for 44 h. Although nuclear maturation did not significantly differ among the four groups, the rate of maturation was significantly higher in the p7 than in the eAFMSC-CMp27 groups (81.7% ± 1.3% vs. 78.9% ± 1.7%, 76.3% ± 1.4%, and 72.0% ± 1.9%, respectively; *p* < 0.05; Figure 3A). The staining intensity of GSH was significantly increased (*p* < 0.05) in the eAFMSC-CMp7 group compared to the other groups, and was essentially the same among the control, p18, and p27 groups. The staining intensity of ROS was significantly decreased in the eAFMSC-CMp7 group compared to all other groups (*p* < 0.05; Figure 3B,C). 

**Figure 4:**
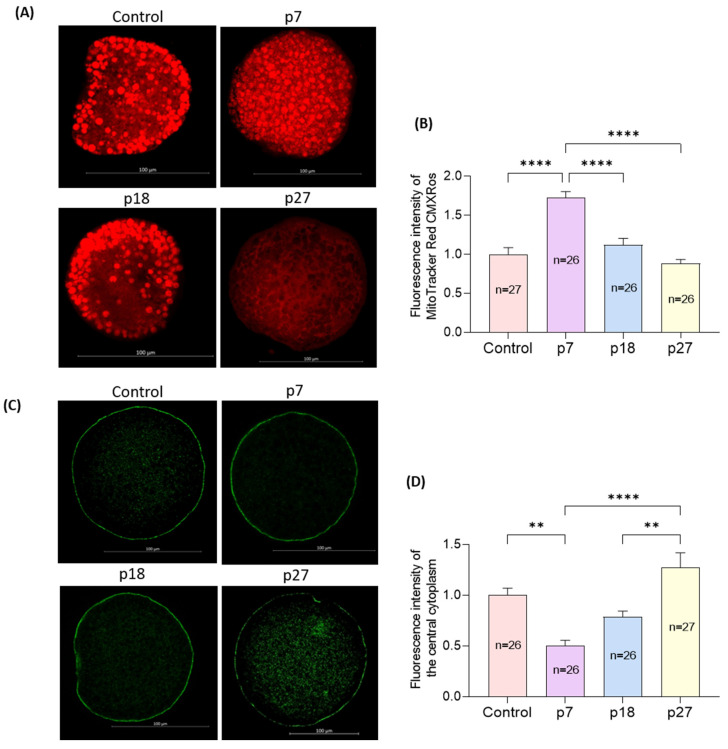
Effects of different passages of eAFMSC-CM on oocyte cytoplasmic maturation. (**A**) Representative images of mitochondrial distribution patterns in porcine oocytes. Control, p18: Heterogeneous, non-aggregated mitochondrial distribution. p7: Homogeneous, non-aggregated mitochondrial distribution. p27: Aggregated mitochondrial distribution. (**B**) Fluorescence intensity of MitoTracker Red CMXRos in MII porcine oocytes. (**C**) Representative images of CGs distribution in porcine oocytes. The CGs of p7 have migrated into the cortical area, and other groups still remained in the central cytoplasm. (**D**) Fluorescence intensity of the central cytoplasm in MII porcine oocytes. The asterisks represent significantly different when compared to the control (**: *p* < 0.01, ****: *p* < 0.0001). Scale bar = 100 μm.

Mitochondrial distribution was heterogeneous throughout the cytoplasm in control oocytes, but not in homogeneous and non-aggregated in eAFMSC-CMp7 oocytes (Figure 4A). The distribution rate of mitochondria was significantly higher in eAFMSC-CMp7 oocytes (*p* < 0.05) than in the other groups, whose distribution did not significantly differ from controls (Figure 4B). Moreover, cortical granules (CGs) were distributed in cortical areas in the eAFMSC-CMp7 group, whereas they were distributed in the central cytoplasm in the other groups (Figure 4C). Significantly more CGs were distributed in oocytes from eAFMSC-CMp7 groups than in those in the control and eAFMSC-CMp27 groups (*p* < 0.05; Figure 4D).

### 2.3. Levels of Antioxidant Biomarkers in Fresh CM and IVM Medium with eAFMSC-CM (Experiment 3)

**Figure 5:**
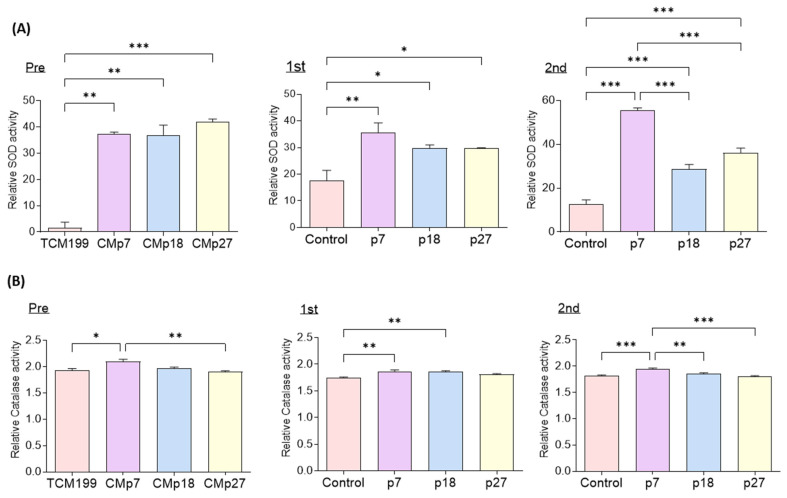
Evaluation of the SOD and catalase activity from IVM medium supernatant in comparison to the control and supplementation with different passage of eAFMSC-CM. (**A**) Superoxide dismutase (SOD) and (**B**) catalase activity. Pre; TCM199 (IVM medium) and Fresh CM. First; Medium after 22 h of IVM. 2nd; Medium after 44 h of IVM. The asterisks represent significantly different when compared to the control (*: *p* < 0.05, **: *p* < 0.01, ***: *p* < 0.001).

We analyzed SOD and CAT activities in fresh CM and IVM culture medium in the four groups after IVM for 22 and 44 h. The SOD and CAT activities were significantly higher in eAFMSC-CMp7 with the pre and first than in the TCM199. The activities of SOD and CAT were significantly higher in the second IVM medium with eAFMSC-CMp7 than those in the control, p18 and p27 groups (55.63 ± 0.93 vs.12.57 ± 1.98, 28.71 ± 2.07, and 36.02 ± 2.21, respectively; *p* < 0.05; Figure 5A; 1.92 ± 0.01 vs. 1.82 ± 0.01, 1.86 ± 0.02, and 1.79 ± 0.02, respectively; *p* < 0.05, Figure 5B).

### 2.4. Effects of eAFMSC-CMp7 during IVM on Porcine Embryo Development after IVF (Experiment 4)

**Figure 6:**
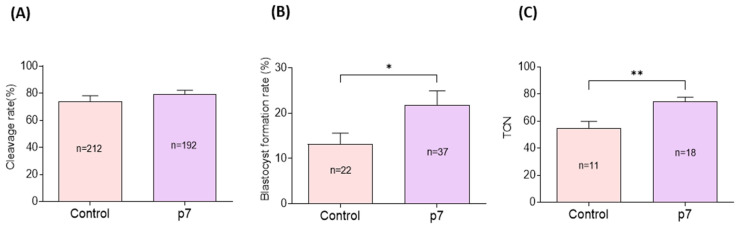
Effects of eAFMSC-CMp7 on porcine embryo development after in vitro fertilization. (**A**,**B**) The cleavage rates and blastocyst formation rates of IVF embryos on Day 2 and Day 6, respectively. (**C**) Total cell number per blastocyst of IVF embryo. The asterisks represent significantly different when compared to the control (*: *p* < 0.05, **: *p* < 0.01).

Cleavage rates did not significantly differ between the eAFMSC-CMp7 group and control group (79.2% ± 2.1% vs. 79.2% ± 3.1% *p* > 0.05; Figure 6A). However, blastocyst formation and the total number of blastocysts were significantly higher in the eAFMSC-CMp7, than in the control group (21.7% ± 3.2% vs. 14.5% ± 2.1%, and 74.4 ± 3.2 vs. 54.7 ± 5.1; *p* < 0.05 for both; Figure 6B,C, respectively). 

**Figure 7:**
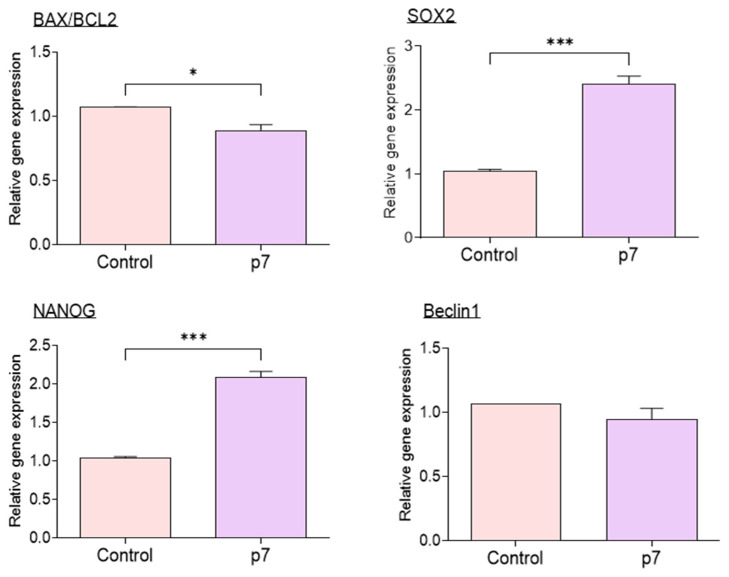
Relative expression of apoptosis-related genes (BAX, BCL2), pluripotency genes (SOX2, NANOG), and autophagy related genes (Beclin1) in blastocysts. The asterisks represent significantly different when compared to the control (*: *p* < 0.05, ***: *p* < 0.001).

The expression of mRNAs associated with apoptosis, pluripotency, and autophagy in IVF blastocysts determined by qRT-PCR revealed a significantly decreased BAX/BCL2 ratio in the eAFMSC-CMp7 group compared to control group (*p* < 0.05; Figure 7), whereas Beclin1 expression did not differ between these groups. The expression of SOX2 and NANOG that are associated with pluripotency significantly differed between the control and eAFMSC-CMp7 groups (*p* < 0.05). 

**Figure 8:**
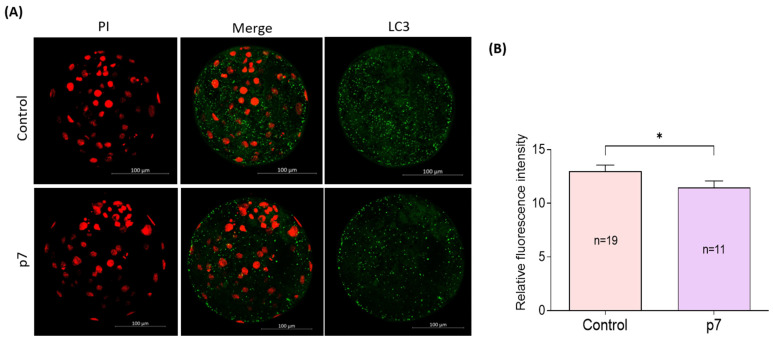
(**A**) Representative fluorescence images show the presence of LC-3 protein in blastocysts. (**B**) Fluorescence intensity of LC3 in blastocysts. The asterisks represent significantly different when compared to the control (*: *p* < 0.05). Scale bar = 100 μm.

The subcellular localization of LC3 of blastocysts was investigated by immunofluorescent staining (Figure 8A). The fluorescence intensity of LC3 is significantly lower in the eAFMSC-CMp7 group than in the control group (11.5% ± 0.61 vs. 13.0% ± 0.58, *p* < 0.05; Figure 8B).

## 3. Discussion

Oocyte developmental competence is affected by direct contact with CCs and by the microenvironment created by the COC during maturation. We found that eAFMSC-CM in porcine IVM medium facilitated oocyte cytoplasmic maturation and embryo development through cumulus cells. 

We initially assessed the effects of eAFMSC-CM passaged 7, 18, and 27 times on IVM to establish the optimal number of passages required for the successful development of in vitro fertilized porcine embryos. Oocyte cytoplasmic maturation was significantly improved in eAFMSC-CMp7 via the expression of genes associated with competent cumulus expansion and cumulus cell apoptosis and improvement in intracellular GSH and ROS, mitochondrial distribution, and CG distribution. We found that eAFMSC-CMp7 significantly improved blastocyst formation, total cell number, and pluripotency-related genes, which are indicators of embryo developmental potential that consequently determine implantation success. In addition, eAFMSC-CMp7 significantly reduced autophagy activity and the expression of associated genes. These results indicated that supplementation of eAFMSC-CMp7 during IVM provides an optimal cytoplasmic maturation microenvironment and preimplantation milieu to develop quality porcine embryos in vitro. 

Communication between oocytes and CCs is important for the acquisition of developmental competence [34,35]. The degree of cumulus cell expansion is routinely used as a gross indicator of oocyte maturation and fertilization in vitro [36]. A decrease in cumulus cell apoptosis increases oocyte maturation capacity and developmental potential [37]. The present study found a significant decrease in the BAX/BCL2 ratio in the eAFMSC-CMp7 group compared to the control group. SOD2 and NRF2 neutralize ROS and protect oocytes and embryos from damage [38]. NRF2 regulates the basal expression of SOD2 and the transcription of genes encoding a variety of antioxidant enzymes [39]. Our results suggested that eAFMSC-CMp7 promotes the expression of antioxidant enzymes in cumulus cells by upregulating NRF2 and SOD2 expression. The mRNA levels of hyaluronan synthase 2 (*HAS2*), *TNFAIP6*, and *PTGS2* genes affiliated with cumulus cell expansion and Cx37 and Cx43 associated with cumulus cell gap junctions were upregulated in porcine cumulus cells in the eAFMSC-CMp7 group. We thus concluded that eAFMSC-CMp7 could provide sufficient bioactive materials through cumulus cells to enhance oocyte competence.

The endogenous antioxidant, GSH, can protect cells from free radicals and ROS, thus playing an important role in the antioxidant defense system of oocytes [40,41]. Excessive ROS generation induced by environmental stress in vitro damages cell structures and activates apoptotic pathways in many types of cells [42]. CCs surrounding oocytes are structurally and metabolically linked to oocytes via gap junctions and they regulate the synthesis and accumulation of GSH in ooplasm [34]. Direct supplementation of antioxidative reagents into the maturation medium improves the GSH content of oocytes [43]. We found that supplementing IVM medium with eAFMSC-CMp7 consistently resulted in significantly generating more GSH and less ROS than the other groups, which indicates powerful antioxidant properties. 

Upon maturation, mitochondria migrate towards the central region, and homogeneous mitochondrial localization is considered a sign of cytoplasmic maturity that is associated with oocyte developmental capacity [44,45,46]. Mammalian CGs are oocyte-specific vesicles located in the subcortical region of fully grown oocytes and function as post-fertilization blocks to polyspermy [47]. Any dysfunction or dislocation of the CGs reduces oocyte competence and affects embryo quality [48]. The ratios of oocytes with mitochondria were higher and the CG distribution was greater in the eAFMSC-CMp7 group, suggesting enhanced cytoplasmic maturation. Mitochondria are critical for energy production via glycolysis and TCA cycle pathways in CCs and as an energy supply for oocyte maturation [49]. Moreover, persistent interaction between CCs and oocytes stabilizes CG distribution, whereas interrupting it induces CG exocytosis that might be responsible for the reduced penetrability of oocytes [50].

Cells use enzymatic antioxidants to resist ROS, with SOD and CAT being the major antioxidants. Intracellular ROS levels in human umbilical cord MSCs are higher at p11 and p17 than at p4, suggesting that senescence occurred according to the cumulative number of passages [51]. Even in human and equine MSCs, successive passages lead to alterations in immune phenotype, differentiation capacity, and cellular senescence [52]. Therefore, we investigated antioxidant biomarker activity in fresh eAFMSC-CM from passages 7, 18, and 27 in the 1st and 2nd IVM media. Our results were consistent with the findings that factors secreted from MSCs into CM contain antioxidants and are predominant elements, and that they exert anti-oxidative effects via paracrine mechanisms [53,54]. Moreover, the findings revealed that oocytes in the second IVM medium supplemented with eAFMSC-CMp7 had significantly more SOD and CAT activities than the other groups. Favorable IVM conditions generated by the release of active antioxidants during early eAFMSC-CM passages might explain the improved oocyte maturation and antioxidant expression in CCs. Next, we used seven passages in subsequent experiments.

We evaluated embryo development and gene expression in oocytes cultured in eAFMSC-CMp7 to further explore this notion. Supplementation with eAFMSC-CMp7 increased anti-apoptotic activities (BCL2) and pluripotency (SOX2 and Nanog) of IVF blastocysts. Moreover, the formation rate and total number of blastocysts significantly improved with eAFMSC-CMp7 through the regulation of apoptotic genes. These results supported our notion that eAFMSC-CM supplementation during the IVM period improves the quality of mature porcine oocytes. 

Various stimuli induce both ROS and autophagy, and the induction of autophagy is antagonized by antioxidants [55,56,57]. The autophagy pathway might be induced under various stress conditions to restore homeostasis as a pro-survival adaptive response to stress. Microtubule-associated light chain protein 3 is a marker of autophagosomes, and changes in its expression reflect levels of autophagy [58,59]. Therefore, autophagy probably protects oocyte maturation under stress. The present findings showed that eAFMSC-CMp7 decreased the intensity of LC3 immunofluorescence emission in porcine blastocysts, indicating reduced autophagy. 

To the best of our knowledge, this is the first study to evaluate the effects of the different passage numbers of eAFMSC-CM on porcine oocytes. We demonstrated that supplementation of eAFMSC-CM during IVM clearly improved oocyte quality and subsequent embryo development. The antioxidant competence of CM from the lower passage of eAFMSC was significantly effective for oocyte cytoplasmic maturation and blastocyst formation. Therefore, eAFMSC-CM would provide an abundant antioxidant-environment influencing the oocyte and embryo developmental competence, and we recommend the usage of eAFMSC-CM from passage 7 for porcine IVM. In a further study, we plan to analyze various factors (many growth factors including cytokines and bioactive factors) that could affect the oocytes in addition to the SOD and CAT investigated in the current study.

## 4. Materials and Methods

### 4.1. Ethics

The Institutional Animal Care and Use Committee (IACUC) of Chungnam National University approved the use of ovaries (Approval no: 202103A-CNU-002). 

### 4.2. Reagents and Chemicals

We purchased all chemicals and reagents from Sigma Chemical Co. (St. Louis, MO, USA), unless otherwise stated.

### 4.3. Preparation of Equine Amniotic Fluid Stem Cell-Conditioned Medium (eAFMSC-CM)

We cultured eAFMSC for 7, 18, and 27 passages until they reached 70% confluence in 100-mm dishes with Dulbecco’s modified Eagle’s medium (DMEM) (Gibco; Thermo Fisher Scientific Inc., Waltham, MA, USA) at 37 °C under 5% CO_2_. The CM was replaced with StemPro^®^ MSC serum-free medium (Gibco; Thermo Fisher Scientific Inc.). After 48 h, media were collected and centrifuged at 300× *g* for 10 min at 4 °C. The supernatant was passed through a 0.22 μM filter and the filtrate was regarded as eAFMSC-CM.

### 4.4. Maturation In Vitro

Porcine ovaries obtained locally within 3 h of slaughter were placed in sterilized 0.9% normal saline at 30 °C. Cumulus oocyte complexes (COCs) were aspirated from antral follicles (3–6 mm) using a 10 mL syringe. Then, COCs with three layers of cumulus cells were selected and cultured at 38.5 °C in a humidified 5% CO_2_ atmosphere in tissue culture medium (TCM)-199 (500 μL) containing 2.5 mM fructose, 0.4 mM L-cysteine, 1 mM sodium pyruvate, 0.13 mM kanamycin, 10% (*v*/*v*) porcine follicular fluid, 10 ng/mL epidermal growth factor (EGF), 10 IU/mL pregnant mare serum gonadotropin (PMSG), and human chorionic gonadotropin (hCG). After 22 h of maturation, the COCs were washed in fresh IVM medium without hormones and cultured for 20–22 h. The maturity of the oocytes was assessed as extrusion of the first polar body in the perivitelline space after denuding. Cumulus cells that separated from the COCs were stored at −80 °C.

### 4.5. Evaluation of Cumulus Cell Expansion 

We quantified degrees of cumulus cell expansion in COCs on a scale of 0–2 after 44 h of IVM as described [60,61]. Degrees of 0, 1, and 2, respectively, indicated no expansion or detachment of cumulus cells from the oocyte, moderate expansion between degrees 0 and 2, and full expansion including the corona radiata.

### 4.6. Detection of Intracellular Glutathione (GSH) and ROS Levels 

Intracellular GSH and ROS levels in oocytes were measured using Invitrogen CellTrackerTM Blue 4-chloromethyl-6, 8-difluoro-7-hydroxycoumarin (CMHC) (Invitrogen, Carlsbad, CA, USA), and Image-iTTM LIVE Green ROS Detection Kits (Invitrogen, Carlsbad, CA, USA), respectively. Oocytes were rinsed three times with North Carolina State University washing medium (NCSU-W) containing 114 mM NaCl, 3.2 mM KCl, 2 mM NaHCO_3_, 0.4 mM NaH_2_PO_4_, 10 mM Na-lactate, 0.5 mM MgCl_2_·6H_2_O, 10 mM HEPES, 2 mM CaCl_2_·2H_2_O, 0.01% polyvinyl alcohol, 12 mM sorbitol, and 0.25 mM Na-pyruvate. Then, GSH levels were determined. The oocytes were incubated for 15 min with 25 μM CellTrackerTM Blue CMF 2 HC dye) at 38.5 °C, washed three times in 5% FBS-PBS (*v*/*v*), and then incubated for 30 min with 25 μM [5-(and 6)-Carboxy-2′,7′-dichlorodihydrofluorescein diacetate] (carboxy-H2DCFDA) at 38.5 °C to measure ROS levels. All oocytes were washed three times after staining and images were visualized by fluorescence microscopy with filters (GSH: 370 nm; ROS: 460 nm). Digital images of oocytes were analyzed with ImageJ software in order to measure the average fluorescence intensity (normalized to the background average intensity).

### 4.7. Assessment of Mitochondrial Distribution

We assessed the mitochondrial distribution in oocytes as follows. Oocytes were washed three times in TCM-199 and incubated for 30 min at 38.5 °C with 250 nM MitoTracker™ Red CMXRos (Thermo Fisher Scientific Inc.). After incubation, they were again washed in TCM-199 and fixed in PBS with 4% paraformaldehyde in phosphate buffered saline (PFA-PBS; *w*/*v*) for 30 min at room temperature (RT). Finally, the oocytes were mounted on glass slides, covered with coverslips, and visualized using an LSM 880 confocal laser scanning microscope (Zeiss, Jena) with filters (between 579 nm and 599 nm). Digital images of oocytes were analyzed with ImageJ software in order to measure the average fluorescence intensity (normalized to the background average intensity).

### 4.8. Assessment of Cortical Granule Distribution 

The distribution of cortical granules in oocytes was measured using fluorescein isothiocyanate (FITC)-labeled peanut agglutinin. Oocytes were fixed in 4% PVA-PBS (*w*/*v*) for 30 min at RT and washed three times with PBS. The oocytes were then permeabilized with 0.5% Triton X-100 for 30 min and rinsed three times with 0.05% Tween-20 (5 min each). Non-specific antigen binding was blocked with 2% BSA in PBS for 1 h, and then the oocytes were incubated with FITC for 2 h at RT. After three washes with 0.05% Tween-20 (5 min each), the sections were mounted on glass slides and examined using the LSM 880 confocal laser-scanning microscope with a filter (488 nm). Digital images of oocytes were analyzed with ImageJ software in order to measure the average fluorescence intensity (normalized to the background average intensity).

### 4.9. Antioxidant Enzyme Activity Assays

SOD and catalase (CAT) activities were quantified in non-conditioned medium (TCM199) and in eAFMSC-CM before and after IVM using colorimetric OxiSelect™ assay kits (Cell Biolabs Inc., San Diego, CA, USA), as described by the manufacturer. The activities of SOD and CAT were assessed by measuring absorbance at 490 and 520 nm, respectively. 

### 4.10. In Vitro Fertilization 

After maturation for 44 h, cumulus cells were removed from the oocytes using 0.1% hyaluronidase. Twenty stage MII oocytes were equilibrated for 20 min in 70-μL droplets of modified Tris-buffered medium (mTBM) containing 113.1 mM NaCl, 3.0 mM KCl, 7.5 mM CaCl_2_·2H_2_O, 20.0 mM Tris Base, 11.0 mM glucose, 5.0 mM Na-pyruvate, 10 μg/mL gentamicin, 1.0 mM caffeine, and 0.2% BSA. For insemination, semen was centrifuged at 500× *g* for 4 min after three washes with Dulbecco’s Phosphate Buffered Saline (DPBS). Then, pelleted sperm was resuspended in 1 mL mTBM and adjusted to the optimal density using mTBM. Thereafter, 20 µL of 2 × 10^6^ sperm/mL were added to the oocytes in 70 uL mTBM droplets and incubated for 6 h in 5% CO_2_ in air at 38.5 °C. Fertilized oocytes were rinsed three times and cultured in 25 µL of porcine zygote medium 3 (PZM-3) containing 10.8 mM NaCl, 0.04 mM MgSO_4_, 1 mM KCl, 0.35 mM KH_2_CO_4_, 0.13 mM kanamycin, 0.2 mM Na-pyruvate, 2.0 mM Ca-lactate, 1.0 mM L-glutamine, 5.0 mM hypotaurine, and 3 mg/mL of BSA at 38.5 °C under an 5% CO_2_ atmosphere for 6 days.

### 4.11. Embryo Development and Total Blastocyst Counts 

Cleavage and blastocyst rates were evaluated at 2 (48 h) and 7 (168 h) days after IVF (Day 0). Depending on the experiment, total blastocysts were counted, RNA was extracted, and the blastocysts were visualized by immunofluorescence staining. The total numbers of blastocysts were determined on day 6. Then, the blastocysts were washed in NCSU-W and immediately stained for 15 min with 5 μg/mL of Hoechst-33342. Blastocysts from each group were mounted on glass slides with a drop of glycerol, covered with coverslip, and evaluated using fluorescence microscopy.

### 4.12. Immunofluorescence Staining

Blastocysts were washed three times with PBS, fixed in 4% PFA-PBS (*w*/*v*) at RT, permeabilized with 0.5% Triton X-100 for 30 min at RT, and rinsed three times with 0.05% Tween-20 (5 min each). Non-specific antigen binding was blocked with 1% BSA in PBS for 1 h, and then the blastocysts were incubated overnight at 4 °C with 1:500-diluted rabbit anti-microtubule-associated protein 1A/1B-light chain 3 (LC3) (ab51520; Abcam, Cambridge, UK). They were then washed three times with 0.05% Tween-20 (5 min each) and labeled with 1:200-diluted Alexa Fluor 488 goat anti-rabbit IGF (ab150077; Abcam, Cambridge, UK) for 1 h at RT. After five washes with 0.05% Tween-20 for 5 min each, blastocysts were counterstained with 10 μg/mL PI for DNA labeling and visualized using the LSM 880 confocal laser-scanning microscope with filter (488 nm). Digital images of oocytes were analyzed with ImageJ software in order to measure the average fluorescence intensity (normalized to the background average intensity).

### 4.13. Analysis of Gene Expression by Quantitative Real-Time PCR 

We extracted RNA from cumulus cells and blastocysts using RNAqueousTM Micro Total RNA Isolation Kits (Invitrogen, USA) as described by the manufacturer. The total RNA concentration was determined using a Biospec-nano spectrophotometer (Shimadzu Corp., Kyoto, Japan). Complementary DNA (cDNA) was synthesized using the Maxime RT premix kit (iN-tRON Biotechnology Inc., Seongnam, Korea) and amplified by Quantitative Real-Time PCR (qRT-PCR) using a CFX96 Touch Real-Time PCR system (Bio-Rad Laboratories Inc., Hercules, CA, USA) as described by the manufacturer, with minor modifications. Each qRT-PCR mixture contained 10 μL SYBR^®^ Green Supermix (Bio-Rad Laboratories Inc.), 0.4 μL (10 pmol/μL) each of forward and reverse primers, 8.2 μL nuclease-free water, and 1 μL cDNA in a PCR plate. The temperature program comprised 40 cycles of 30 s at 95 °C, 30 s at 55 °C, and 30 s at 72 °C. The expression of each target gene in the CCs and blastocysts was quantified relative to that of the housekeeping gene, glyceraldehyde 3-phosphate dehydrogenase (GAPDH), using the equation: R = 2^−[ΔCt sample−ΔCt control^. Table 1 shows the primers included in the reactions.

### 4.14. Experimental Design 

This study was divided a control group without CM and three groups with eAFMSC-CM obtained after 7, 18, and 27 passages (p7, p18, and p27) as follows; (1) Control: 100% TCM-199, (2) 50% TCM-199 + 50% eAFMSC-CMp7, (3) 50% TCM-199 + 50% eAFMSC-CMp18, and (4) 50% TCM-199 + 50% eAFMSC-CMp27. Conditioned medium was added to the IVM medium during the maturation period of 44 h. We analyzed the effects of eAFMSC-CM on the degree of cumulus cell expansion and the expression of mRNAs associated with cumulus expansion, apoptosis, antioxidant enzymes, and gap junctions in CCs (Experiment 1), and on oocyte cytoplasmic maturation, ROS and GSH levels, and the distribution of mitochondria and cortical granules in oocytes (Experiment 2). We evaluated antioxidant enzymes in culture supernatants derived from the four groups before (TCM 199 and eAFMSC-CM), and after 22 (first IVM medium) and 44 (second IVM medium) h of IVM using ELISA (Experiment 3). We also investigated embryo development, autophagic activity, apoptosis, pluripotency, and genes associated with autophagy in IVF blastocysts derived from oocytes in eAFMSC-CMp7 (Experiment 4).

### 4.15. Statistical Analysis 

All data generated from at least triplicate experiments were statistically analyzed using GraphPad Prism 9.0.0 (GraphPad, San Diego, CA, USA) and are presented as means ± SEM. All data from experiments 1–3 were evaluated using a univariate analysis of variance (ANOVA) with Tukey tests. All data from experiment 4 were evaluated using Student t-tests and the results are presented as means ± standard deviation. Values were considered statistically significant at * (*p* < 0.05), ** (*p* < 0.01), *** (*p* < 0.001), and **** (p < 0.0001).

## 5. Conclusions

This study demonstrated that eAFMSC-CM treatment acts as an antioxidant in the optimal passage of eAFMSC during IVM of porcine oocyte. Moreover, eAFMSC-CM improves oocyte quality and embryonic development after in vitro fertilization by reducing ROS generation and increasing GSH levels during meiosis. These findings provide evidence that eAFMSC-CM would provide an abundant antioxidant-environment influencing the oocyte and embryo developmental competence, and we recommend the usage of eAFMSC-CM from passage 7 for porcine IVM.

## Figures and Tables

**Table 1:** Specific primers used for Real-Time PCR.

Gene	Sequence (5′-3′)	Product Size (bp)	NCBI Accession No.
GAPDH	F: gtc ggt tgt gga tct gac ct	207	NM_001206359.1
	R: ttg acg aag tgg tcg ttg ag		
BAX	F: ggt cgc gct ttt cta ctt tg	111	XM_003127290
	R: cga tct cga agg aag tcc ag		
BCL2	F: aaa caa tgc agc agc tga ga	85	NM_214285
	R: aac cac ccc agc tag agt ca		
HAS2	F: cag gga caa ttc agc cac tt	100	NM_214053.1
	R: ggt gac atg ttg gga gct tt		
TNFAIP6	F: gaa gca cgg tcg ggc aag	141	NM_001159607.1
	R: cat cca ccc agc agc aca g		
PTGS2	F: tcc tga aca cct ccg ctt tg	147	NM_214321.1
	R: agc cgt tca tcg tcc cat tc		
Cx37	F: cac cct gtc cct acc tcg ta	101	NM_001244224.1
	R: gag cac cag gga gat gag tc		
Cx43	F: gct ggt cgt atc ctt ggt gt	83	NM_001244212.1
	R: tct ttc cct tca cac gat cc		
SOD2	F: tgg agg cca cat caa tca ta	136	NM_214127.2
	R: agc ggt caa ctt ctc ctt ga		
NRF-2	F: gtg cct ata agt ccc ggt ca	108	MH101365.1
	R: atg cag agc ttt tgc cct ta		
BECN1	F: gat agt ggc gga aaa tct cg	160	NM_001044530.1
	R: cat ctg ggc ata acg cat ct		
SOX2	F: cgc aga cct aca tga acg	103	NM_001123197.1
	R: tcg gac ttg acc act gag		
NANOG	F: ccc gaa gca tcc att tcc ag	171	NM_001129971.1
	R: tgt gga aga atc agg gct gt		

## Data Availability

The original contributions presented in the study are included in the article. Further inquiries can be directed to the corresponding authors.
